# Genome-wide identification and expression profiling of the U-box gene family in poplar under salt stress

**DOI:** 10.3389/fpls.2026.1767843

**Published:** 2026-02-13

**Authors:** Zhi Dong, Jun Li, Yinchen Gong, Shaoran Li, Tiandong Wu, Yibo An, Lian Luo

**Affiliations:** 1 Chongqing Forestry Investment Development Co. Ltd., Chongqing, China; 2 National Reserve Forest Research Institute, Chongqing, China; 3 Chongqing Key Laboratory of Forest Ecological Restoration and Utilization in the Three Gorges Reservoir Area, Chongqing, China; 4 Chongqing Key Laboratory of Tree Germplasm Innovation and Utilization, Chongqing, China; 5 China National Forestry Group Co., Ltd., Beijing, China; 6 Northeast Forestry University, Harbin, China

**Keywords:** gene family evolution, genome-wide analysis, *Populus*, stress response, U-box gene family

## Abstract

**Introduction:**

The U-box gene family, defined by the presence of a conserved U-box domain, participates in various biological processes, particularly in plant responses to abiotic stress. However, a comprehensive analysis of this gene family in *Populus trichocarpa* has not yet been conducted. Investigating the characteristics of U-box genes in poplar will deepen our understanding of the molecular mechanisms underlying salt tolerance and provide a theoretical basis for enhancing its environmental adaptability.

**Methods:**

In this study, we performed a genome-wide identification and characteristic analysis of the U-box gene family. Analyses included phylogenetic relationships, gene structures, conserved motifs, promoter cis-acting elements, chromosomal distribution, gene duplication events, and synteny with other plant species. In addition, the expression patterns of selected U-box genes under salt stress were validated using quantitative real-time PCR (qRT-PCR).

**Results:**

In this study, 103 U-box genes were identified in the *P. trichocarpa* genome and mapped to 18 chromosomes. These genes encode proteins with molecular weights ranging from 9.5 to 129.3 kDa and isoelectric points between 4.31 and 9.08, with most predicted to localize in the nucleus. Promoter analysis revealed numerous cis-acting elements associated with development, abiotic stress responses, growth regulation, and hormone signaling. Collinearity analysis identified 4 tandem and 30 segmental duplication events. Transcriptome data showed that 40 and 21 *PtrPUBs* were differentially expressed in roots and leaves, respectively, under salt stress.

**Discussion:**

These findings suggest that U-box genes may play important roles in regulating poplar’s salt stress response. Overall, this study provides a theoretical foundation and valuable genetic resources for the identification of salt tolerance genes in poplar.

## Introduction

Plants are frequently exposed to abiotic stresses, among which high salt stress posing a serious threat to their growth and development. Salt stress leads to cellular dehydration, membrane damage, and impaired function of membrane-bound enzymes, resulting in metabolic disruption and altered plasma membrane permeability (Zhou et al., 2024). Excessive accumulation of salt ions further induces osmotic and oxidative stress, disturbing plant metabolism and energy homeostasis (van Zelm et al., 2020). Additionally, salt stress interferes with sugar signaling and alters the levels of key sugars, such as sucrose, fructose, and glycolytic intermediates (Zhao et al., 2021). These disruptions negatively affect photosynthesis, inhibit cell division and proliferation, and ultimately suppress overall plant growth and biomass accumulation (Zhao et al., 2021). To cope with salt-alkali stress, plants have evolved diverse physiological and molecular mechanisms, including the regulation of Na^+^ and K^+^ uptake and transport, activation of antioxidant enzymes to scavenge reactive oxygen species (ROS), and enhancement of osmotic adjustment capacity (Agarwal et al., 2013). Under salt stress, plants rapidly accumulate osmolytes such as proline, betaine, and organic acids, which contribute to osmotic balance and mitigate cellular damage (Rao et al., 2023). Moreover, salt stress induces the expression of a wide range of stress-responsive genes involved in osmoprotectant biosynthesis, ion transport, antioxidant defense, and signal transduction, which collectively enhance salt tolerance by modulating metabolic pathways (Kumar et al., 2021). These complex stress-response processes require precise regulation of gene expression and protein turnover. Among them, protein ubiquitination, a key post-translational regulatory mechanism, plays a central role in stress signal transduction and adaptive responses (Karthik et al., 2025). Proteins encoded by the U-box gene family function as critical executors of this regulatory pathway.

In this regard, the U-box gene family, encoding a major class of E3 ubiquitin ligases, has emerged as a critical regulator of plant stress responses. The U-box gene family encodes proteins characterized by the presence of a conserved U-box domain, most of which function as E3 ubiquitin ligases that confer substrate specificity in the ubiquitination pathway (Trenner et al., 2022). This family includes both HECT- and RING-type E3 ligases (Trenner et al., 2022). U-box proteins are widely distributed in plants and play crucial roles in cell cycle regulation, signal transduction, morphogenesis, protein degradation, secretion, and responses to environmental stimuli (Mao et al., 2022). They have been implicated in various developmental processes, including root and reproductive organ formation (Yee and Goring, 2009). For example, in *Arabidopsis*, *PUB25* and *PUB26* are key regulators of floral organ development (Li et al., 2021). In rice, loss of *OsPUB33* function enhances photosynthesis and sugar transport, promoting cell proliferation and grain filling (Li et al., 2021). Mutation of *OsPUB15* disrupts primary root formation, leading to stunted seedling growth or lethality (Wang et al., 2015). In wheat, *TaPUB1* negatively regulates the abscisic acid (ABA) signaling pathway through interaction with TaPYL4 and TaABI5, thereby influencing seed development (Zhang et al., 2021b).

Furthermore, U-box genes are also involved in abiotic stress responses. In poplar, overexpression of *PalPUB79* enhances drought tolerance by targeting *PalWRKY77* for ubiquitination and degradation, thus positively regulating ABA signaling (Tong et al., 2021). Under salt stress, PUB30 mediates the degradation of HB24, downregulating *SWEET11* expression, reducing sucrose availability, inhibiting root growth, and contributing to salt stress adaptation (Wang et al., 2023). In wheat, *TaPUB1* modulates ion transporter activity to maintain Na^+^/K^+^ homeostasis and enhances antioxidant enzyme activity to mitigate oxidative stress, thereby improving salt tolerance (Wang et al., 2020). Additionally, *TaPUB1* has been shown to regulate cadmium uptake and tolerance by modulating the stability of TaIRT1 and TaIAA17 proteins (Zhang et al., 2021a). PUB35 negatively regulates ABA signaling via AFP1-mediated degradation of ABI5, playing a role in salinity and osmotic stress responses (Li et al., 2024).

The U-box domain was first identified in the yeast protein UFD2, comprising a conserved domain of approximately 70 amino acids, marking it as the first known U-box protein (Hatakeyama and Nakayama, 2003). Subsequently, U-box family genes have been identified in various plants, including cotton (Lu et al., 2020), wheat (Yang et al., 2021) and pear (Wang et al., 2021). Recently, Liu et al. (2025) reported a genome-wide characterization of the U-box gene family in *Populus alba* and analyzed their transcriptional responses to abiotic stresses, providing an important reference framework for PUB genes in poplar. However, the regulatory roles of PUB genes in salt stress, particularly in *Populus simonii* × *P. nigra*, remain largely unexplored. In this study, a comprehensive genome-wide identification of *PUB* genes in poplar was conducted, accompanied by analyses of cis-regulatory elements and gene expression patterns under salt stress conditions. Furthermore, the evolutionary relationships and gene structures of the *PUB* family were systematically examined. These findings provide a theoretical basis and valuable genetic resources for the identification of candidate genes associated with salt stress tolerance in poplar.

## Materials and methods

### Identification of poplar U-box gene family members

The whole-genome data of *Populus trichocarpa* v3.1 were obtained from the Phytozome database. The hidden Markov model (HMM) profile of the U-box domain (PF04564) was retrieved from the Pfam database (http://pfam.xfam.org/). Candidate U-box proteins were identified by searching the *P. trichocarpa* protein database using HMMER v3.0 with an E-value cutoff of 1 × 10^−5^ (Finn et al., 2011). Redundant sequences were removed, and all putative U-box proteins were further verified using the SMART (http://smart.embl-heidelberg.de/) and Pfam databases to confirm the presence of a complete U-box domain. The molecular weight and isoelectric point of each candidate protein were predicted via the ExPASy ProtParam tool (http://web.expasy.org/protparam/), and subcellular localization was predicted using ProtComp 9.0 (http://linux1.softberry.com).

### Phylogenetic analysis of poplar U-box proteins

Protein sequences of the poplar U-box family were downloaded from Phytozome 12. Multiple sequence alignment was conducted using ClustalW (Thompson et al., 2002). A phylogenetic tree was constructed with MEGA11 software using the neighbor-joining (NJ) method and visualized through the EvolView online tool.

### Analysis of gene structure, conserved motifs, and promoter elements

Gene structures were analyzed using the GSDS online tool (http://gsds.cbi.pku.edu.cn/). Conserved motifs were identified with MEME (http://meme-suite.org/tools/meme) using default parameters. Promoter sequences comprising 2000 bp upstream of each U-box gene were extracted from Phytozome 12. Cis-acting regulatory elements were predicted with PlantCARE (http://bioinformatics.psb.ugent.be/webtools/plantcare/html/). Visualization was performed using TBtools software (Chen et al., 2020).

### Chromosomal localization, homology, and gene duplication analysis

Chromosomal positions of U-box genes were mapped using data from Phytozome 12. Gene duplication events were identified using MCScanX (Wang et al., 2012). Collinearity between poplar U-box genes and homologs from other species (eucalyptus, tomato, *Arabidopsis thaliana*, maize, rice, and potato) was analyzed using the Dual Synteny Plotter tool, with results visualized in TBtools (Chen et al., 2020).

### Plant material and stress treatments

The hybrid poplar of *Populus simonii* × *P. nigra*, was used in this study, and transcriptome data were obtained from published work (Cheng et al., 2021a). Differentially expressed genes (DEGs) were identified with the |log2(FC)|>1 and false discovery rate (FDR)<0.05 using the DESeq package from Bioconductor (version 1.12.1). Seedlings were cultured on half-strength Murashige and Skoog (1/2 MS) medium. One-month-old seedlings were treated with 0 mM (control) or 150 mM NaCl for 0, 12 and 24 hours, respectively. Samples of roots and leaves from the stressed plant materials at different time points were collected using liquid nitrogen. Total RNA was extracted using a TAKARA RNA extraction kit, and cDNA was synthesized using a TAKARA reverse transcription kit. Expression levels of key salt-responsive genes were determined by quantitative real-time PCR (qRT-PCR) using a fluorescence quantitative PCR kit. Each qRT-PCR reaction was performed with three technical replicates for each biological replicate. Relative gene expression was calculated using the 2^^(−ΔΔCt)^ method. All primers used in this study are listed in **Supplementary Table S7**.

## Results

### Analysis of amino acid sequence characteristics of poplar U-box proteins

**Figure 1:**
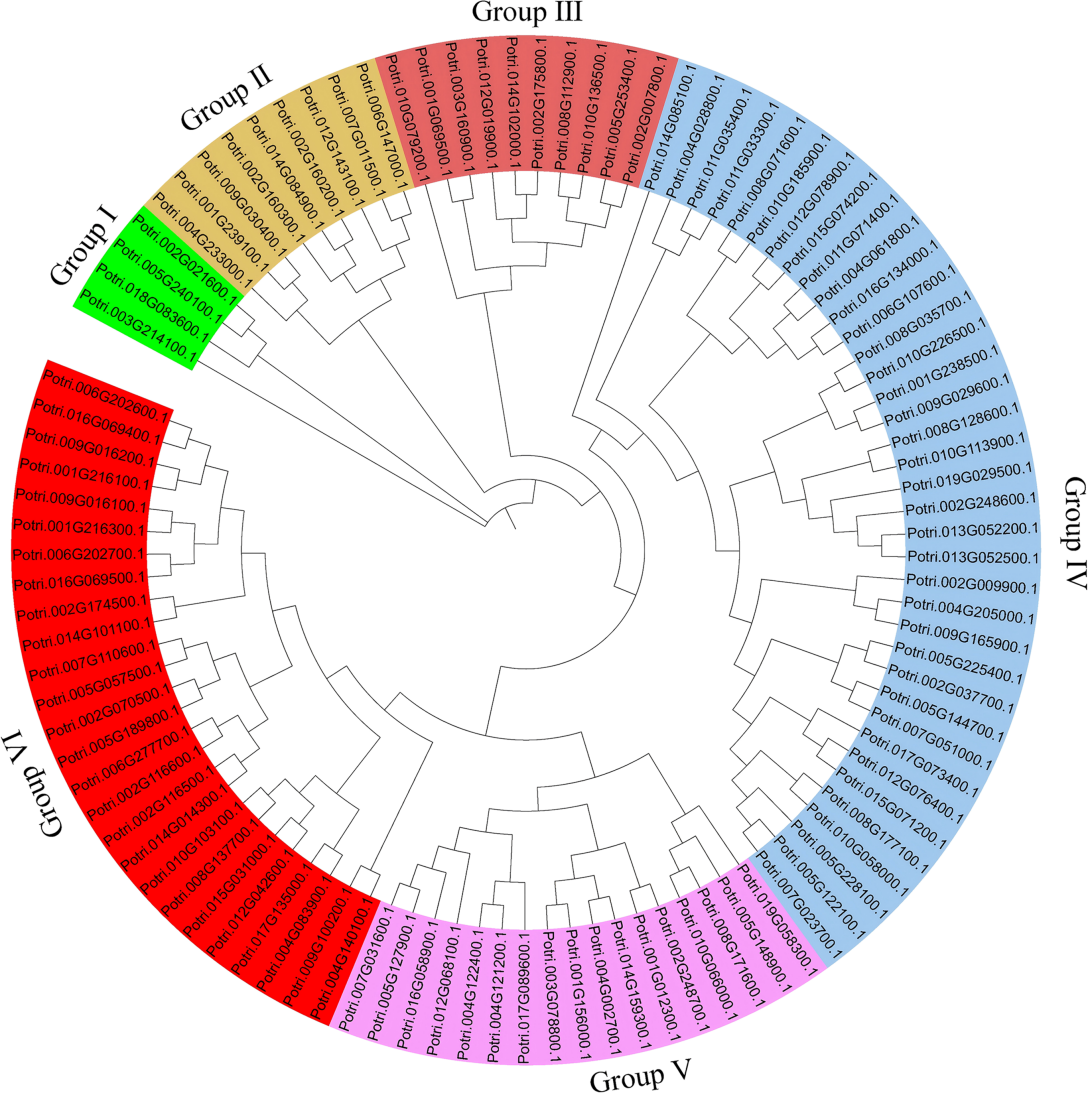
Phylogenetic analysis of poplar U-box proteins. The phylogenetic tree was constructed using the neighbor-joining method in MEGA11. The U-box proteins were classified into six groups, with each group represented by a different color.

The physicochemical properties of the poplar U-box family proteins were analyzed (**Supplementary Table S1**). The protein with the highest molecular weight was Potri.005G240100.1 (129,258.26 Da), while the smallest was Potri.014G085100.1 (9,518.8 Da). The predicted isoelectric points (pI) ranged from 4.31 to 9.08, with 54 U-box proteins exhibiting pI values below 7, indicating that most U-box proteins are acidic. The aliphatic index varied between 54.02 and 117.4. Twenty-six proteins had a positive hydropathicity index, classifying them as hydrophobic, whereas 69 proteins were hydrophilic, with a negative hydropathicity index. Subcellular localization prediction indicated that 87 U-box proteins localize to the nucleus, six to both the cytoplasm and nucleus, and one (Potri.005G148900.1) exclusively to the cytoplasm. Phylogenetic analysis of the 103 poplar U-box proteins grouped them into six distinct clades (**Figure 1**). Group IV was the largest group with 37 members, followed by Group VI (26), Group V (17), Group III (10), Group II (9), and Group I (4), the smallest group.

### Conserved motif characterization of poplar U-box proteins

**Figure 2:**
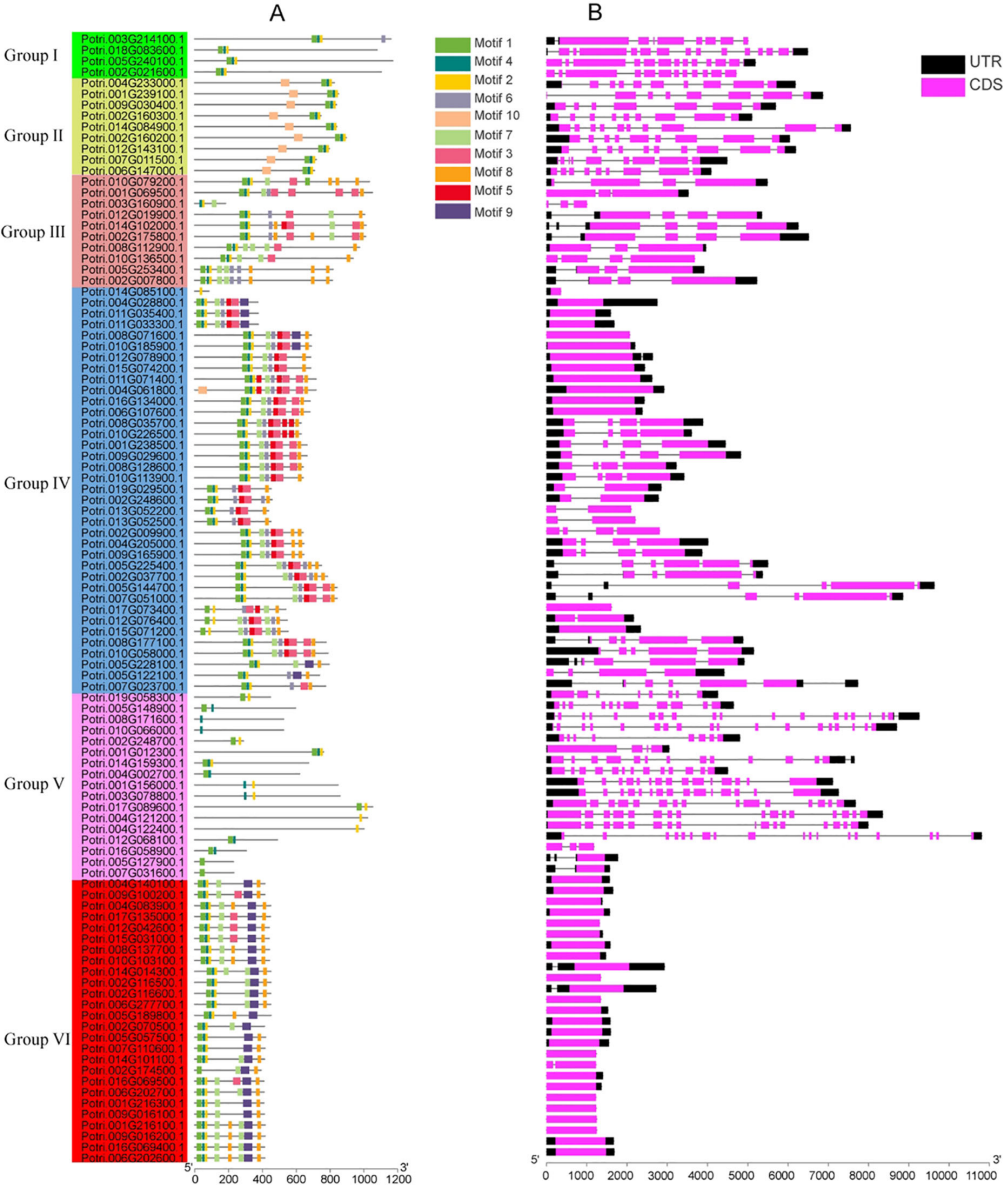
Analysis of gene structures, conserved protein domains, and motifs of poplar U-box genes. **(A)**, Gene structure analysis showing exon-intron organization. **(B)**, Conserved motif analysis of U-box proteins. Different motifs are represented by colored boxes.

Motif analysis identified ten conserved motifs across the U-box gene family. While motif composition varied among different groups, members within the same clade generally exhibited similar motif patterns (**Figure 2A**). For instance, members of Group I shared motifs 1, 2, and 4; Group II contained motifs 1, 2, 4, and 10; and Group VI included motifs 1, 8, and 9. Motif 1 was present in nearly all PUB members and corresponded to the conserved U-box domain, which is essential for E3 ubiquitin ligase activity. The remaining motifs showed clade-specific distribution patterns, suggesting their potential roles in protein-protein interactions and substrate recognition. Gene structure analysis revealed substantial variation in exon–intron organization. As illustrated in the **Figure 2B**, 36 genes lacked introns, 8 genes contained a single intron, and 55 genes possessed more than two introns. Members of the same subfamily typically exhibited conserved exon–intron structures, whereas significant structural divergence was observed between subfamilies. Notably, the Group VI subfamily contained relatively few introns, while the Group V subfamily had a higher number of introns.

### Promoter sequence analysis of poplar U-box genes

**Figure 3:**
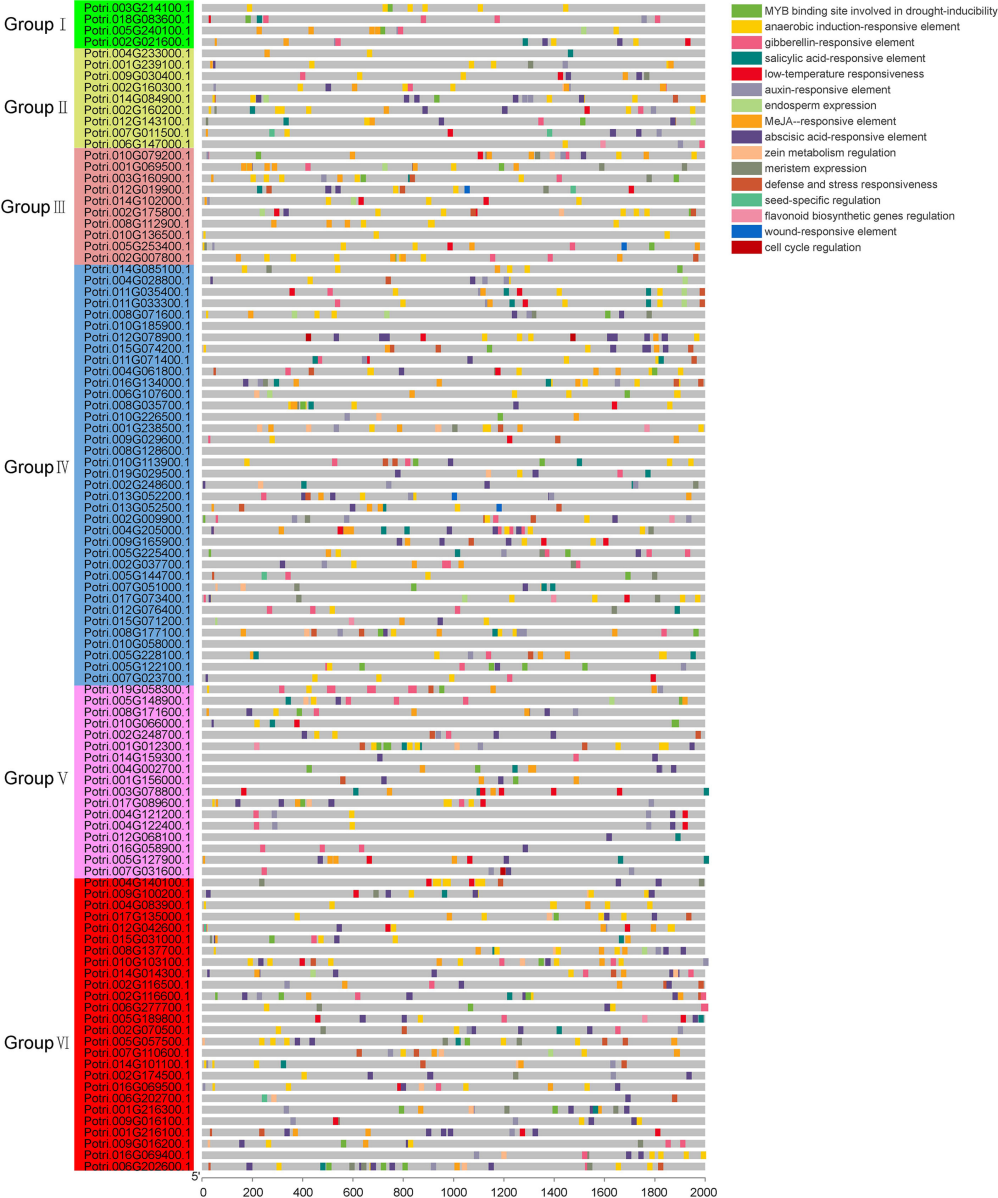
Cis-regulatory element analysis of poplar U-box gene promoters. The colored ellipses on the right denote different types of cis-elements involved in various biological processes.

The 2000 bp promoter regions upstream of the poplar U-box genes were analyzed using PlantCARE to identify cis-regulatory elements. Based on functional annotations, the identified elements were classified into three categories: abiotic stress-responsive elements, growth and development-related elements, and hormone-responsive elements (**Figure 3**; **Supplementary Table S2**). Abiotic stress-related elements included MYB-binding sites associated with drought response, low-temperature response elements, and cis-elements involved in dehydration and salt stress. Elements linked to growth and development were associated with meristem-specific expression, seed-specific regulation, and cell cycle control. Hormone-responsive elements were involved in signaling pathways related to methyl jasmonate (MeJA), gibberellin, and abscisic acid (ABA). The widespread occurrence of these elements suggests that U-box genes may play important roles in regulating poplar growth and development and mediating responses to abiotic stress.

### Chromosomal localization of poplar U-box genes

**Figure 4:**
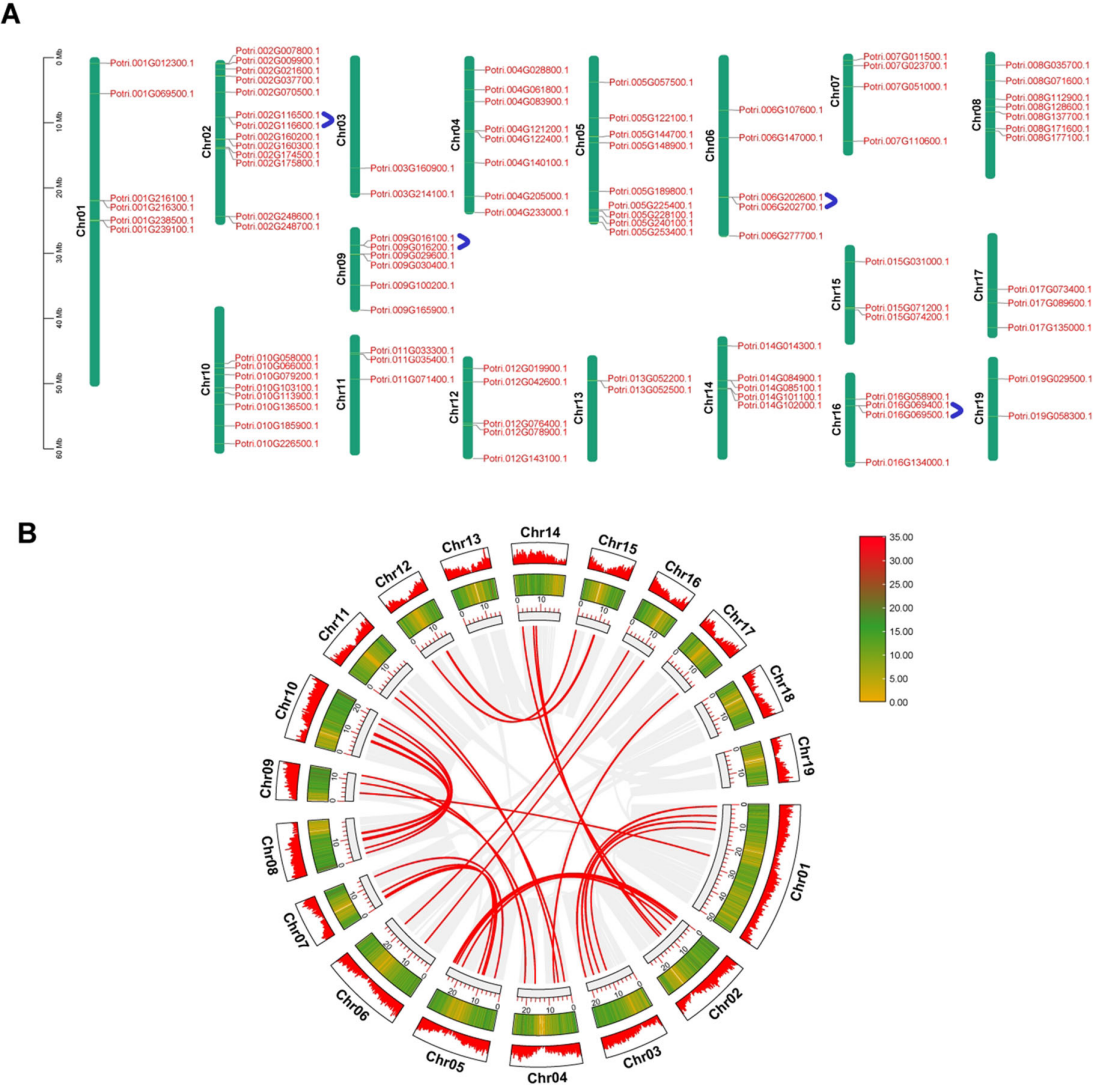
Chromosomal distribution and duplication events of poplar U-box genes. **(A)**, Chromosomal localization of U-box genes. Blue arcs represent different tandemly duplicated gene pairs. **(B)**, Segmental duplication analysis. Red lines represent segmentally duplicated gene pairs; gray lines indicate homologous blocks in the Populus genome. The outer ring and heatmap indicate gene density on each chromosome.

Using chromosomal annotation data, the 103 identified U-box genes were mapped onto the poplar genome (**Figure 4A**). Their distribution across chromosomes was uneven: chromosome 2 contained the highest number of U-box genes (13), followed by chromosome 5 (9 genes), and chromosomes 4 and 10 (8 genes). The remaining chromosomes harbored between 1 and 7 U-box genes, while chromosome 18 lacked any U-box genes. MCScanX analysis identified four tandem duplication events involving eight genes located on chromosomes 2, 6, 9, and 16. Additionally, 30 segmental duplication events were detected and distributed across multiple chromosomes (**Figure 4B**; **Supplementary Table S3**). Notably, most of these collinear genes belong to Group IV and Group VI subfamilies, comprising 24 and 16 genes, respectively. The relatively high number of genes in Group IV and Group VI further supports the hypothesis that gene duplication contributed significantly to the expansion of the U-box gene family in poplar.

### Collinearity analysis of poplar U-box family genes

**Figure 5:**
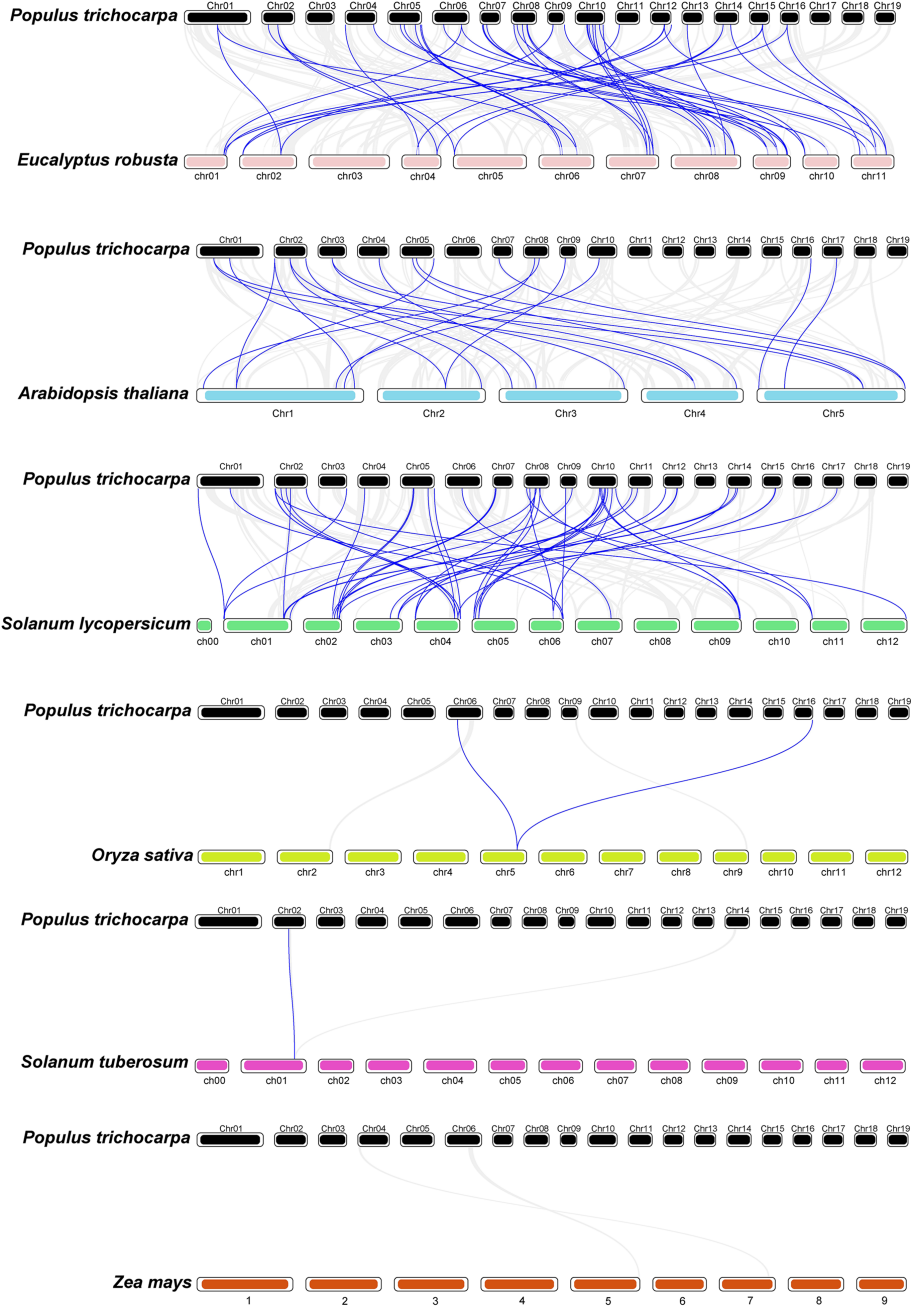
Collinearity analysis of U-box genes among six species including poplar. Blue lines represent homologous U-box gene pairs between poplar and other species. Gray lines indicate orthologous gene pairs across species.

To investigate the evolutionary relationships of poplar U-box genes, synteny analyses were conducted with homologous genes from four dicot species (*Eucalyptus*, *Arabidopsis thaliana*, *Solanum lycopersicum*, and *Solanum tuberosum*) and two monocot species (*Zea mays* and *Oryza sativa*). A total of 45 duplication events were identified with *Eucalyptus*, 41 with tomato, 21 with *Arabidopsis*, one with potato, two with rice, and none with maize (**Figure 5**). Moreover, several *PtrPUB* genes were found to have homologs across multiple species. For instance, three genes (*Potri.008G171600.1*, *Potri.010G058000.1*, and *Potri.005G122100.1*) share homologs in *Eucalyptus*, *Arabidopsis*, and *Solanum tuberosum*, while *Potri.002G160200.1* is conserved in *Eucalyptus*, *Arabidopsis*, and *Zea mays*. Additionally, seven genes are shared between *Eucalyptus* and *Arabidopsis*, and six genes are shared between *Eucalyptus* and *Solanum tuberosum* (**Supplementary Figure S1**, **Supplementary Table S4**).

### Spatiotemporal expression patterns of *PtrPUB* genes in different tissues

**Figure 6:**
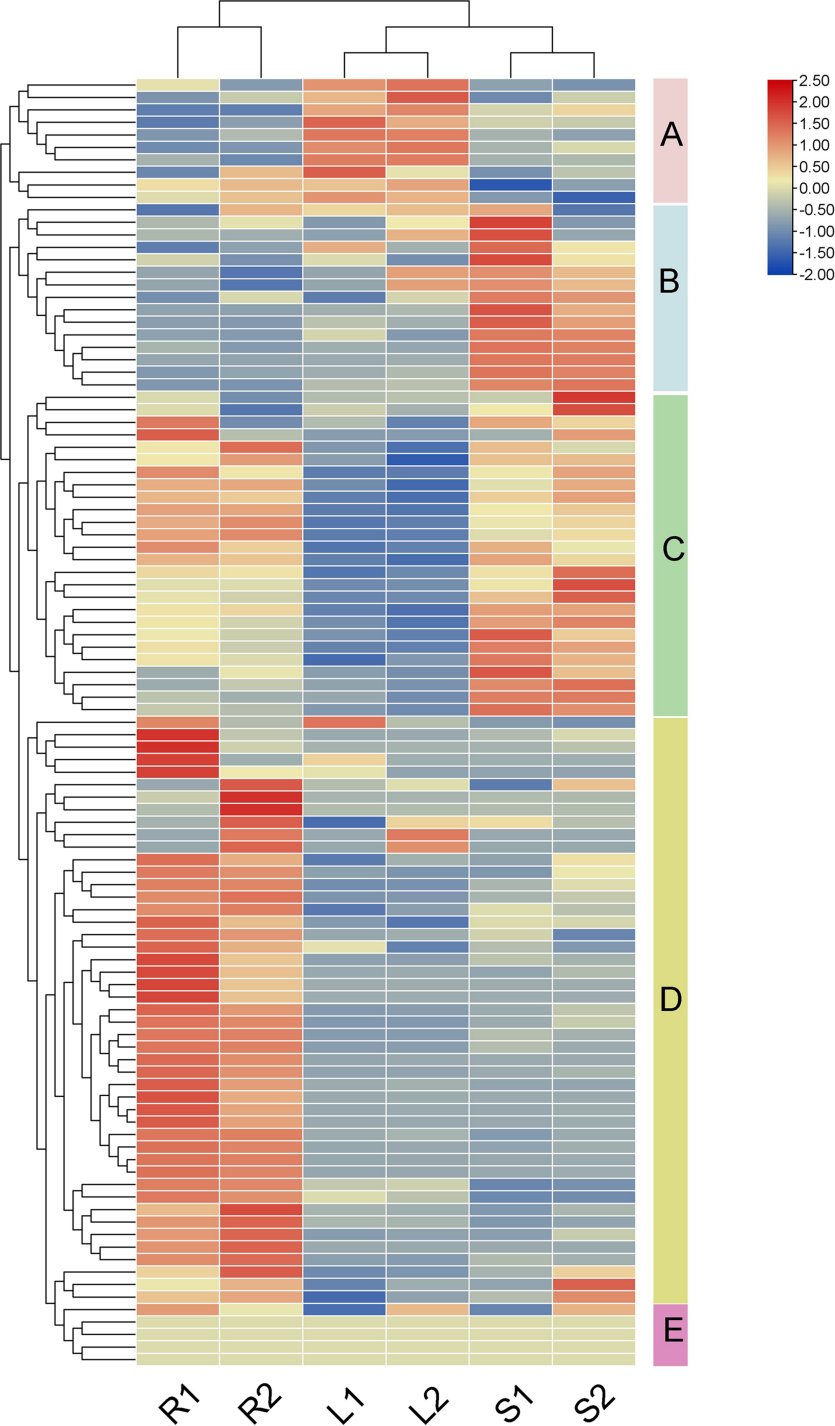
Expression patterns of poplar U-Box genes across different tissues. R, L, and S represent roots, leaves, and stems under non-salt-stress conditions, respectively.

Understanding the expression patterns of genes across various tissues is essential for elucidating their biological functions in plant growth and development. To investigate the tissue-specific expression characteristics of *PtrPUB* genes, we conducted an expression analysis of 103 *PtrPUB* genes and generated a heat map (**Figure 6**; **Supplementary Table S5**). Based on tissue-specific expression patterns, these genes were categorized into five groups: cluster A was predominantly expressed in leaves, cluster B in stems, cluster C in both roots and stems, cluster D in roots, and cluster E showed no significant expression differences among tissues. These results indicate that *PtrPUB* genes exhibit distinct spatiotemporal expression specificity, suggesting their potential involvement in tissue-specific physiological regulation.

### Identification of key salt-tolerance U-box genes in poplar

**Figure 7:**
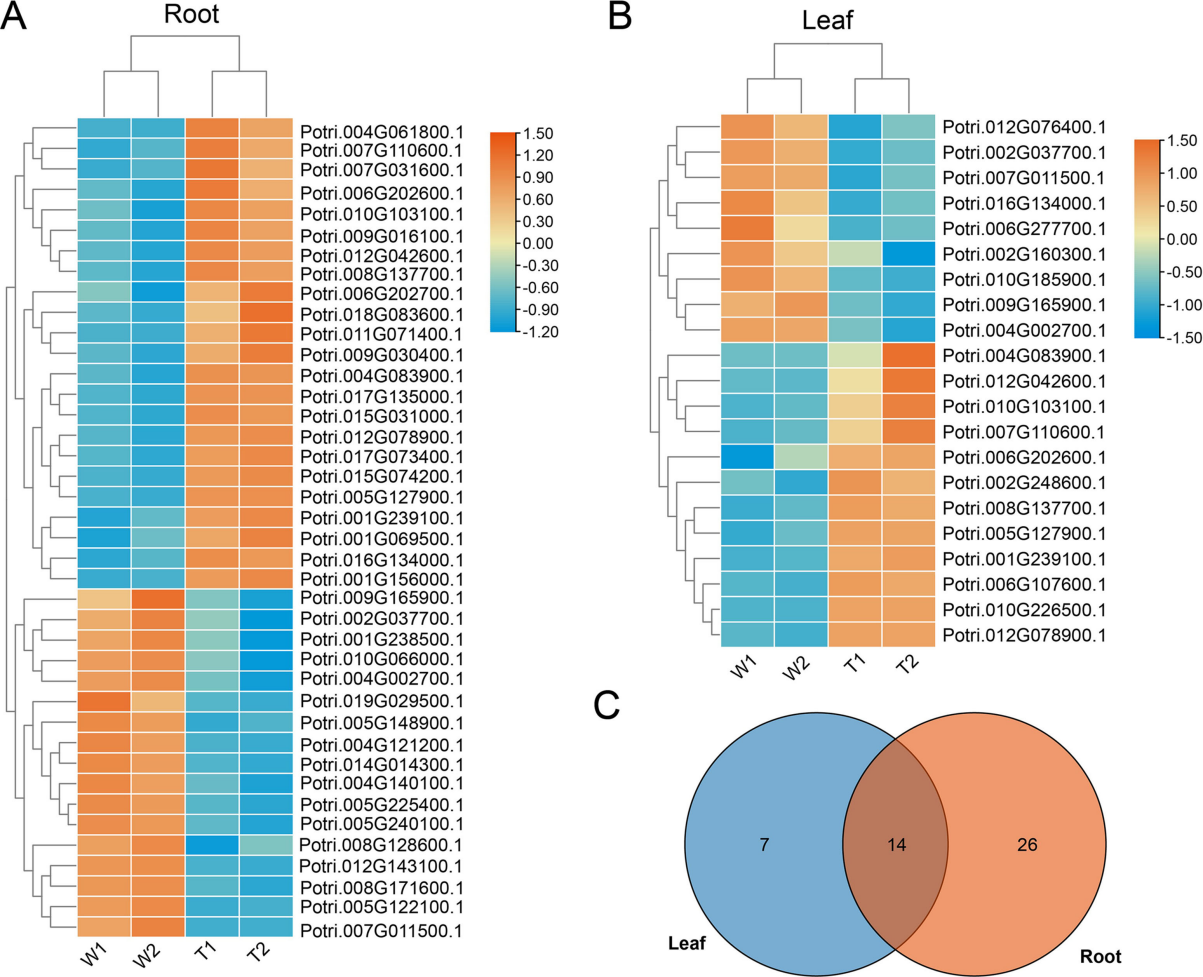
Expression patterns of *PtrPUB* genes under salt stress. **(A, B)**, Heatmaps showing DEGs in leaves and roots under control (W) and salt stress (T). **(C)**, Venn diagram illustrating the overlap of DEGs between the two tissues.

RNA-Seq data were used to assess the expression profiles of U-box genes under salt stress in different poplar tissues. The heatmap clearly categorized differentially expressed genes into upregulated and downregulated groups (**Figure 7**; **Supplementary Table S6**). In roots, 40 differentially expressed genes were identified, including 23 upregulated and 17 downregulated genes (**Figure 7A**). In leaves, 21 differentially expressed genes were found, with 12 upregulated and 9 downregulated (**Figure 7B**). Notably, 14 genes were identified as differentially expressed genes in both leaves and roots (**Figure 7C**).

### Expression analysis of *PtrPUB* genes under salt stress

**Figure 8:**
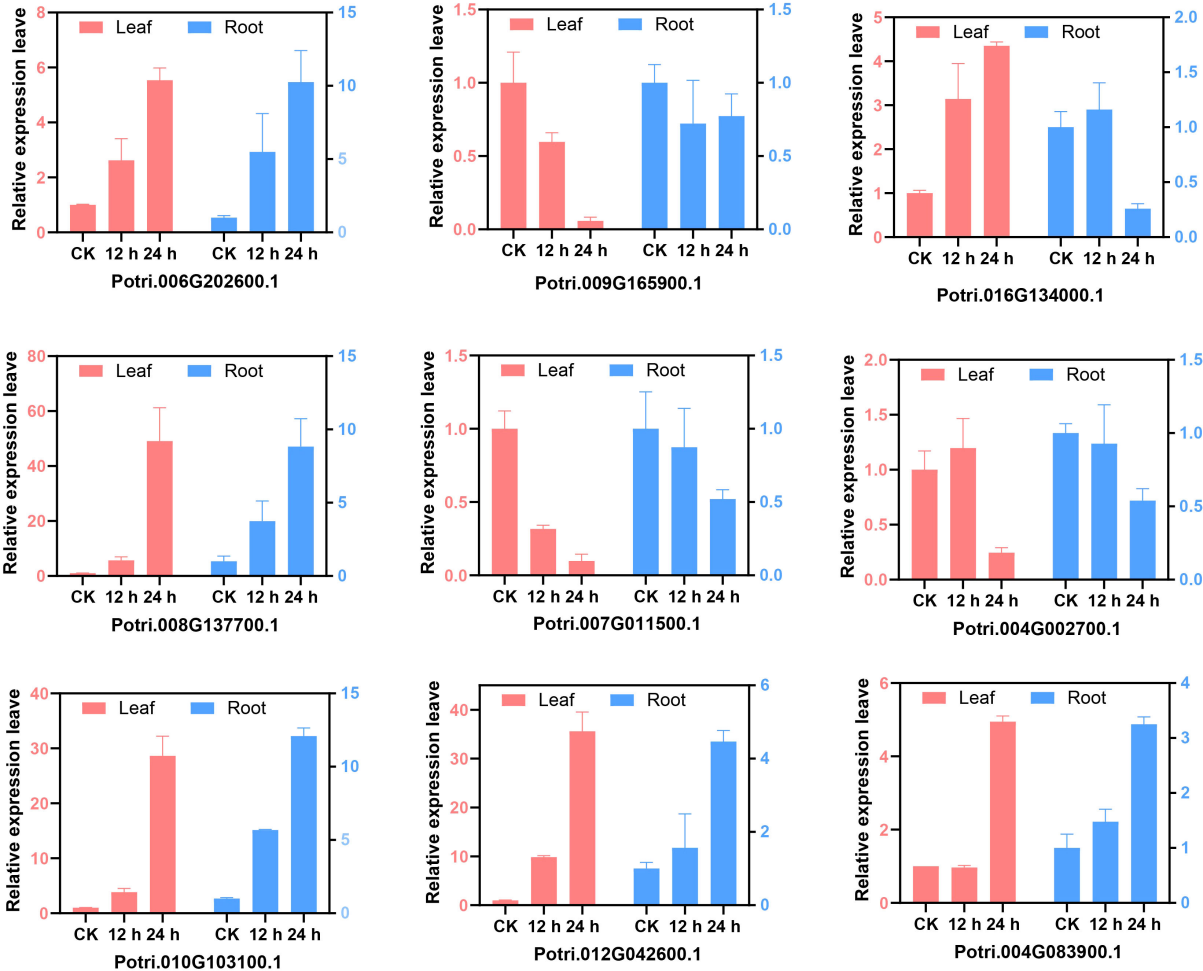
Expression levels of *PtrPUB* genes in two tissues under salt stress. One-month-old poplar seedlings were treated with 150 mM NaCl for 0, 12, and 24 h. Gene expression was normalized to the reference gene Actin and calculated using the 2^^(−ΔΔCt)^ method. Data represent the mean ± SD of three independent biological replicates.

Based on RNA-seq data from salt-stressed poplars, nine differentially expressed genes were selected from leaf and root tissues for further analysis (**Figure 8**). Their expression levels were examined in leaves and roots following NaCl treatment at 12 and 24 hours. The results revealed that six genes were significantly upregulated in both tissues after NaCl exposure, while three genes were significantly downregulated. These expression patterns were consistent with the transcriptome data, indicating that these genes may play roles in poplar’s response and tolerance to salt stress.

## Discussion

The U-box gene family is broadly distributed in plants and plays a critical role in regulating growth and development, particularly under abiotic stress conditions (Trenner et al., 2022). In this study, 103 U-box genes were identified in the poplar genome and classified into six subfamilies. Notably, members of Group VI possess fewer introns, which may facilitate more efficient gene expression tailored to specific physiological functions. Conversely, Group V members contain a higher number of introns, potentially enabling more complex regulatory mechanisms and contributing to functional diversity. Motif analysis revealed nine conserved motifs with potential functional implications. Motif 1 corresponds to the conserved U-box domain responsible for E3 ubiquitin ligase activity, while other motifs likely contribute to substrate recognition, protein-protein interactions, or regulatory specificity (Van Roey et al., 2014). Furthermore, the presence of a subset of U-box proteins extracellularly indicates potential additional roles outside the nucleus, possibly in cell signaling or interactions with the cell wall and extracellular matrix (Trujillo, 2018). Similar localization patterns have been observed in cabbage, supporting the notion that such distribution is conserved among plant species (Wang et al., 2023). This conserved subcellular targeting underscores the functional versatility of U-box proteins, allowing them to participate in diverse cellular pathways critical for plant growth and adaptation.

The evolution of gene families is largely driven by DNA segmental duplications, tandem duplications, and gene conversion events, all of which contribute to genetic diversification and adaptive potential under stress conditions (Flagel and Wendel, 2009). Our analysis revealed that segmental duplication (28 pairs) was far more prevalent than tandem duplication (4 pairs) within the U-box gene family, indicating that segmental duplication is the primary mechanism driving its expansion in poplar. This phenomenon was also observed in the late embryogenesis-abundant (Cheng et al., 2021a) and xyloglucan endotransglucosylase/hydrolase (Cheng et al., 2021b) gene families of poplar. Furthermore, collinearity analysis with six other species demonstrated that poplar U-box genes share more syntenic gene pairs with dicotyledonous species than with monocots, suggesting greater evolutionary conservation within dicots. These duplicated genes likely contributed to the functional diversification of the U-box gene family during evolution.

Cis-acting elements in promoter regions are critical regulators of gene expression (Cui et al., 2023). Characterization of these elements enables the identification of genes involved in developmental and stress response pathways (Marand et al., 2023). We found that the promoter regions of poplar U-box genes are enriched with MYB binding sites associated with drought responsiveness, gibberellin response elements, endosperm expression motifs, and abscisic acid (ABA) response elements (Zhang et al., 2025). The abundance of these cis-elements indicates a significant involvement of U-box genes in hormone signaling pathways and stress responses. Moreover, the presence of shoot- and root-specific elements, cell cycle regulatory elements, and seed-specific motifs suggests that U-box genes may modulate poplar’s adaptation to abiotic stress by influencing developmental processes.

The U-box gene family plays a vital role in the development of plant tissues and organs and exhibits distinct tissue expression patterns. For example, in *Coffea canephora*, 40 genes are expressed in leaves, 33 in stems, and 14 in fruit tissues (Liu et al., 2024). Tomato U-box genes exhibit strong expression in meristems and leaf tissues, implicating their role in vegetative growth (Sharma and Taganna, 2020). In the present study, a tissue-specific expression analysis of 103 *PpPUB* genes revealed distinct spatiotemporal expression patterns in roots, stems, and leaves. Based on transcript levels, these genes were classified into five expression groups, underscoring their potential involvement in tissue-specific physiological processes. Similar expression patterns have also been observed in *Setaria italica* (Zhou et al., 2024).These findings provide valuable insights into the functional diversity of *PpPUB* genes during poplar growth and development.

U-box family genes play critical roles in plant responses to abiotic stress. For example, MYB-related elements in twelve *SbPUB* genes in sorghum regulate flavonoid biosynthesis, a key pathway contributing to salt tolerance (Cui et al., 2023). In contrast, overexpression of *GmPUB8* in soybean leads to hypersensitivity to salt and drought stresses (Wang et al., 2016), highlighting the diverse functional roles of U-box genes across species. In this study, 40 differentially expressed *PtrPUB* genes were identified in roots and 21 in leaves under salt stress, with 14 genes co-expressed in both tissues. Functional annotation based on homologous *Arabidopsis* genes suggests that several poplar *U-box* genes may be involved in developmental regulation during stress. For example, *AT3G52450* (homologous to *Potri.010G103100.1*) and *AT2G35930* (homologous to *Potri.006G202600.1*) participate in water stress responses and negatively regulate PAMP-triggered immunity (Trujillo et al., 2008). *AT1G60190* (homologous to *Potri.012G078900.1*) and *PUB18* are implicated in repressing salt stress responses, thereby modulating salt tolerance (Bergler and Hoth, 2011). *AT2G23140* (homologous to *Potri.002G037700.1*) is broadly expressed and regulates root development, pollen tapetum formation, and ROS-mediated chloroplast degradation (Wang et al., 2013). Additionally, *AT2G45910* (homologous to *Potri.001G239100.1*) promotes seed germination and flower development (Cao et al., 2006). Collectively, these findings underscore the pivotal role of U-box genes in coordinating plant stress responses and developmental processes.

## Conclusion

In summary, we identified 103 U-box gene family members in poplar, distributed across 18 chromosomes. Promoter analyses revealed abundant cis-acting elements related to abiotic stress response, growth, development, and hormone signaling. Collinearity analysis indicated four pairs of tandem duplications and 30 pairs of segmental duplications, highlighting the evolutionary dynamics of this gene family. RNA-Seq data revealed differential expression of U-box genes under salt stress, with 40 and 21 genes showing significant regulation in roots and leaves, respectively. These results collectively suggest that poplar U-box genes play essential roles in mediating responses to salt stress. This study provides a valuable theoretical foundation and gene resource for future efforts aimed at identifying and characterizing salt tolerance genes in poplar.

## Data Availability

The original contributions presented in the study are included in the article/**Supplementary Material**. Further inquiries can be directed to the corresponding authors.

## Supplementary material

Supplementary Figure 1 Upset plot diagram of the poplar U-box genes throughout diverse species. The yellow color represents the number of genes that have collinearity between poplar and other species, the black circles connected by line segments represent genes that are shared by different species, and the black column represents the number of shared genes.

Supplementary Table 1 The list of 103 U-box genes identified in this article

Supplementary Table 2 The list of cis-regulatory elements of *PtrPUB* gene promoters.

Supplementary Table 3 The list of repetitive events in poplar U-box genes.

Supplementary Table 4 The homologous relationships between the poplar U-box genes and the other species.

Supplementary Table 5 Tissue-specific expression analysis of the *PtrPUB* gene.

Supplementary Table 6 *PtrPUB* gene expression analysis under salt stress.

Supplementary Table 7 The primers sequences used in this study.
