# Supplementary figures and images for: Genome-wide identification and expression profiling of the U-box gene family in poplar under salt stress
Source: Front Plant Sci. 2026 Feb 13;17:1767843. doi: 10.3389/fpls.2026.1767843 (PMC12946096; doi:10.3389/fpls.2026.1767843)

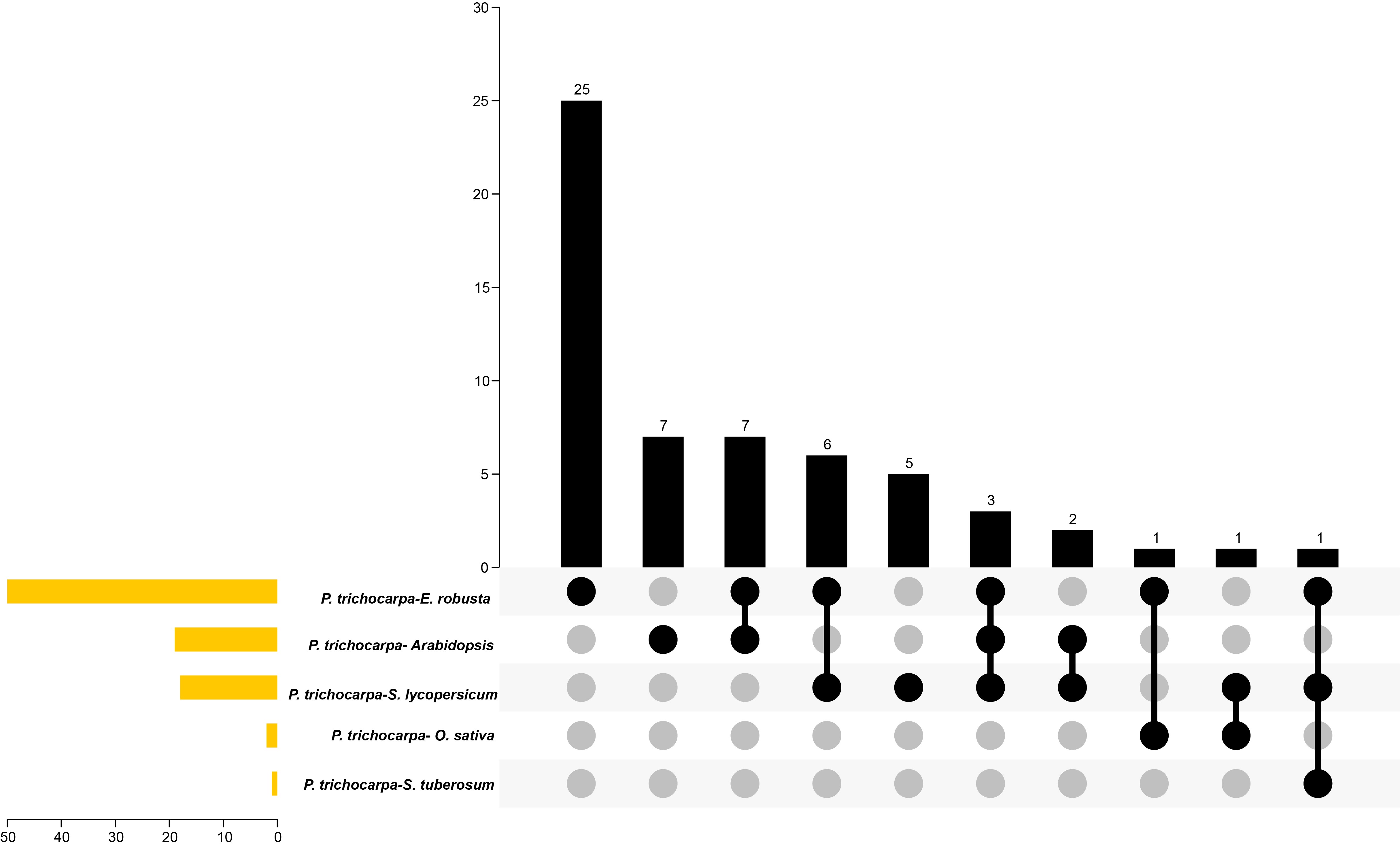

Supplement: Supplementary Figure 1 — Upset plot diagram of the poplar U-box genes throughout diverse species. The yellow color represents the number of genes that have collinearity between poplar and other species, the black circles connected by line segments represent genes that are shared by different species, and the black column represents the number of shared genes. [file Image1.jpeg]
